# Temporal trends in intracerebral hemorrhage: Evidence from the Austrian Stroke Unit Registry

**DOI:** 10.1371/journal.pone.0225378

**Published:** 2019-11-20

**Authors:** Christoph Schellen, Alexandra Posekany, Julia Ferrari, Stefan Krebs, Wilfried Lang, Michael Brainin, Dimitre Staykov, Marek Sykora, on behalf of the Austrian Stroke Unit Registry Collaborators

**Affiliations:** 1 Department of Radiology, Rudolf Foundation Hospital ("Krankenanstalt Rudolfstiftung"), Vienna, Austria; 2 Department for Clinical Neurosciences and Preventive Medicine, Danube University, Krems, Austria; 3 Department of Neurology, St. John's Hospital, Vienna, Austria; 4 Medical Faculty, Sigmund Freud University, Vienna, Austria; 5 Department of Neurology, St. John's Hospital, Eisenstadt, Austria; 6 I. Department of Neurology, Comenius University, Bratislava, Slovakia; University of Münster, GERMANY

## Abstract

**Background:**

To assess changes in frequency, severity, complications, therapy and outcome of intracerebral hemorrhage in patients treated in stroke units in Austria, we evaluated data from the Austrian Stroke Unit Registry between 2008 and 2016.

**Methods and findings:**

Data of 6707 cases of ICH covering a time span of 9 years and including information on age, risk factors, pre-stroke modified Rankin Score (mRS), baseline stroke severity (NIHSS), complications, therapy, functional outcome, and mortality were extracted from the Austrian Stroke Unit Registry. A multivariate regularized logistic regression model and linear models for temporal dependence were computed for analyzing statistical inference and time trends. Bonferroni correction was applied to correct for multiple testing.

Between 2008 and 2016, the proportion of ICH admissions to stroke units in Austria declined, with a shift among patients towards older age (>70 years, p = 0.04) and lower admission NIHSS scores. While no significant time trends in risk factors, pre-stroke mRS and medical complications were observed, therapeutic interventions declined significantly (p<0.001). Three-month mortality increased over the years independently (p = 0.003).

**Conclusions:**

Despite declining incidence and clinical severity of ICH we observed a clear increase in three-month mortality. This effect seems to be independent of predictors including age, admission NIHSS, pre-morbid MRS, or medical complications. The observations from this large retrospective database cohort study underline an urgent call for action in the therapy of ICH.

## Introduction

Intracerebral hemorrhages (ICH) make up approximately 10–20% of all strokes in western high-income countries, however, the burden of stroke mortality and morbidity associated with ICH is disproportionately high [1–5]. While the incidence of ischemic stroke decreased in recent decades [2,6,7], limited data exists on the incidence of ICH and available reports came to conflicting results [1,2,6–12]. Mortality in patients with ICH remains high. The one-year mortality rate in ICH is approximately 55% [4,7]. One patient in three will die within the first month following ICH onset, with nearly 50% of the deaths occurring within the first 48 hours [1,2,4,5,7]. While some authors reported declining mortality following ICH in recent years [3,6,10], others found a decrease only in younger patients (<75 years of age) [12] or observed decreasing 30-day mortality but no changes within the first 48 hours from stroke onset [2]. Numerous studies, including a large meta-analysis by van Asch in 2010 that reached back into the 1980s, observed no change in mortality after ICH [1,7–9,11,13]. The aim of this analysis was to examine trends in ICH incidence, severity, complications, therapy and outcome of ICH from 2008 to 2016 in subjects registered in the Austrian Stroke Unit Registry (ASUR) [14–16].

## Methods

Data of 6707 cases of ICH covering a time span of 9 years and including information on age, risk factors, pre-morbid modified Rankin Score (mRS), baseline stroke severity (National Institutes of Health Stroke Scale, NIHSS), therapy, complications, functional outcome and mortality were extracted from the Austrian Stroke Unit Registry. The subjects were divided into 4 age groups (18 to 50 years, >50 to 70 years, >70 to 80 years, and >80 years) and admission NIHSS scores were split into 4 categories (low 0–5, moderately low 6–10, moderately high 11–20, and high 21–42). The functional outcome at 3 months was dichotomized into favorable outcome (mRS 0–3), reflecting a broad degree of independence in the daily activities of life, versus unfavorable outcome (mRS 4–6), indicating considerable dependency or death. The distribution of the data was visualized using histograms and graphs with 95% pointwise standard confidence intervals for population proportions. Exploratory time trend analyses were computed with a standard linear model approach. Multivariate regularized logistic regression models (least absolute shrinkage and selection operator ("LASSO") regression, fitted by applying version 2.0–16 of the R package "glmnet") were fitted for analyzing statistical relations and temporal trends. Bonferroni correction was applied to correct for multiple testing. All statistics were performed using statistical software R 3.3.3 [17].

### Standard protocol approvals, registrations, and patient consents

The Austrian Stroke Unit Registry is part of a governmental quality assessment program for stroke care in Austria financed by the Federal Ministry of Health. It is based on the federal law promoting quality in healthcare ("Gesundheitsqualitätsgesetz"). Anonymized data are centrally administered by the Gesundheit Österreich GmbH, and scientific analyses are approved and supervised by an academic review board [18].

## Results

Out of 119785 stroke patients ≥ 18 years of age who were recorded in the Austrian Stroke Unit Registry between 2008 and 2016, 6707 patients (5.6%; 53% male; median age 74.5 years, interquartile range (IQR) 18.0 years, range 19.1–102.1 years) were treated for ICH. In the descriptive analyses, the relative proportion of ICH patients in the general study population (all strokes) declined significantly over time (6.3% in 2008 vs 5.8% in 2012 vs 4.7% in 2016, Fig 1). While the sex distribution remained unchanged at the 5% significance level, there was a significant transition towards older patients in time trend analyses. The percentage of patients aged >70 to 80 years and >80 to 120 years increased while the proportion of patients aged >50 to 70 years declined over the years. The fraction of patients aged 18 to 50 years remained unchanged (Fig 2).

**Fig 1:**
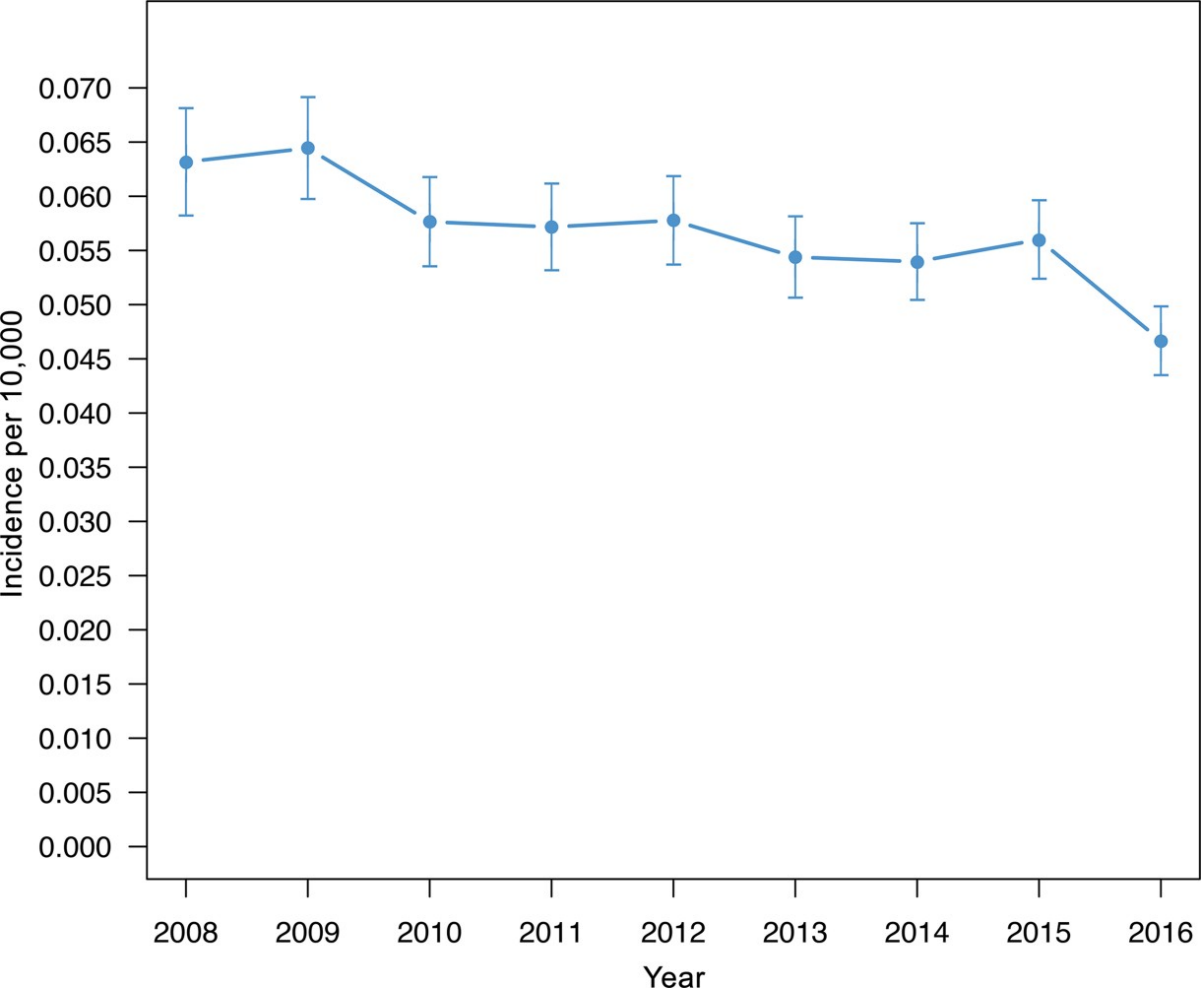
Time trend in intracerebral hemorrhage incidence. Time trend of intracerebral hemorrhage incidence in stroke patients registered in the Austrian Stroke Unit Registry between 2008 and 2016.

**Fig 2:**
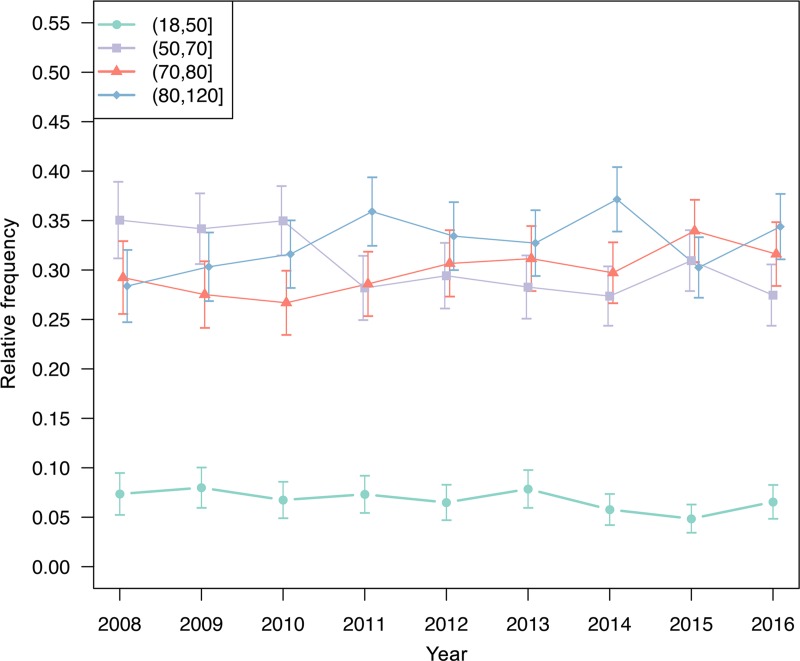
Age distribution over time. Age distribution over time in stroke patients registered in the Austrian Stroke Unit Registry between 2008 and 2016.

### Admission stroke severity and pre-morbid functional status

**Fig 3:**
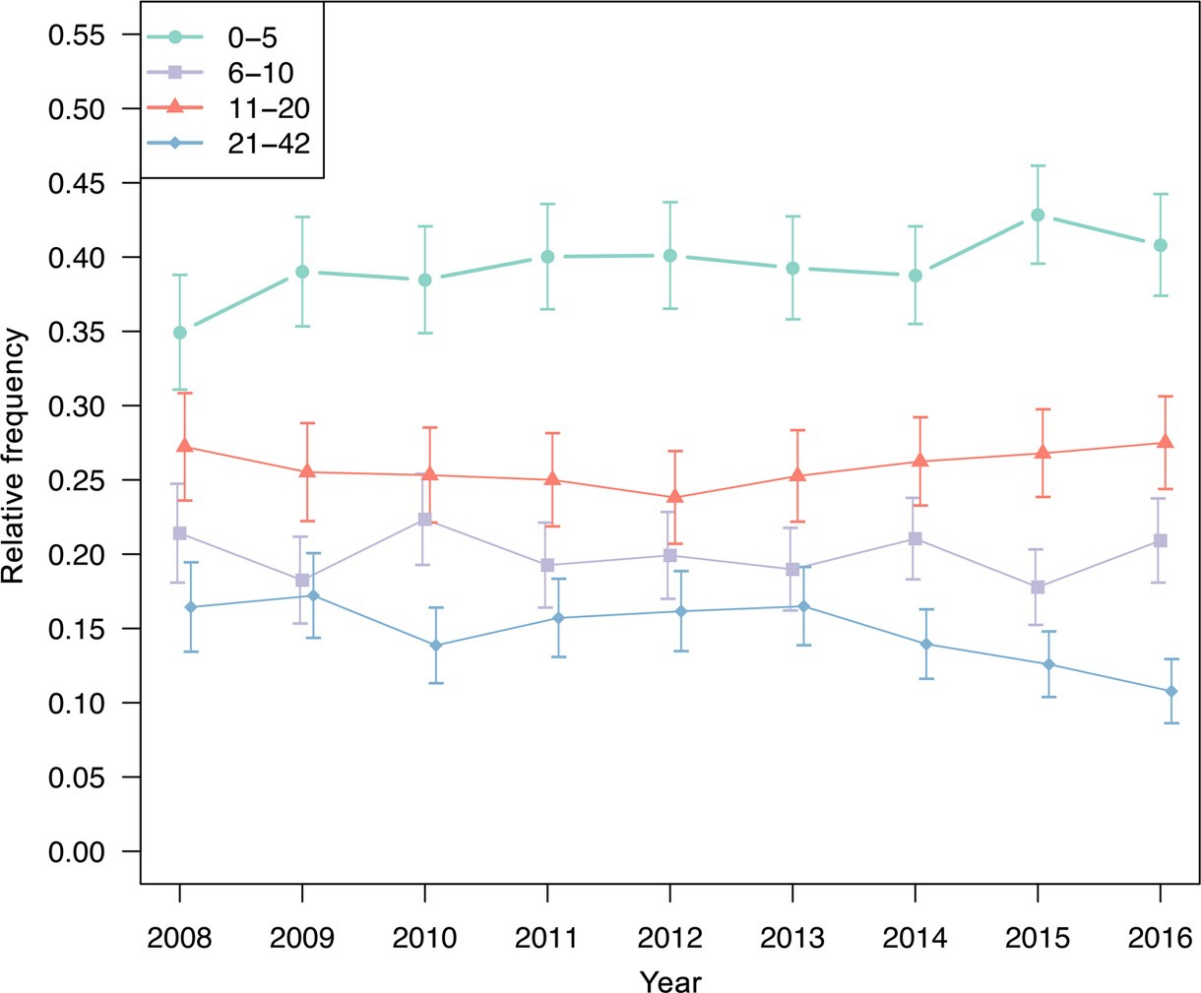
Time trend in admission NIHSS scores. Time trend of admission National Institutes of Health Stroke Scale (NIHSS) scores in stroke patients registered in the Austrian Stroke Unit Registry between 2008 and 2016.

**Fig 4:**
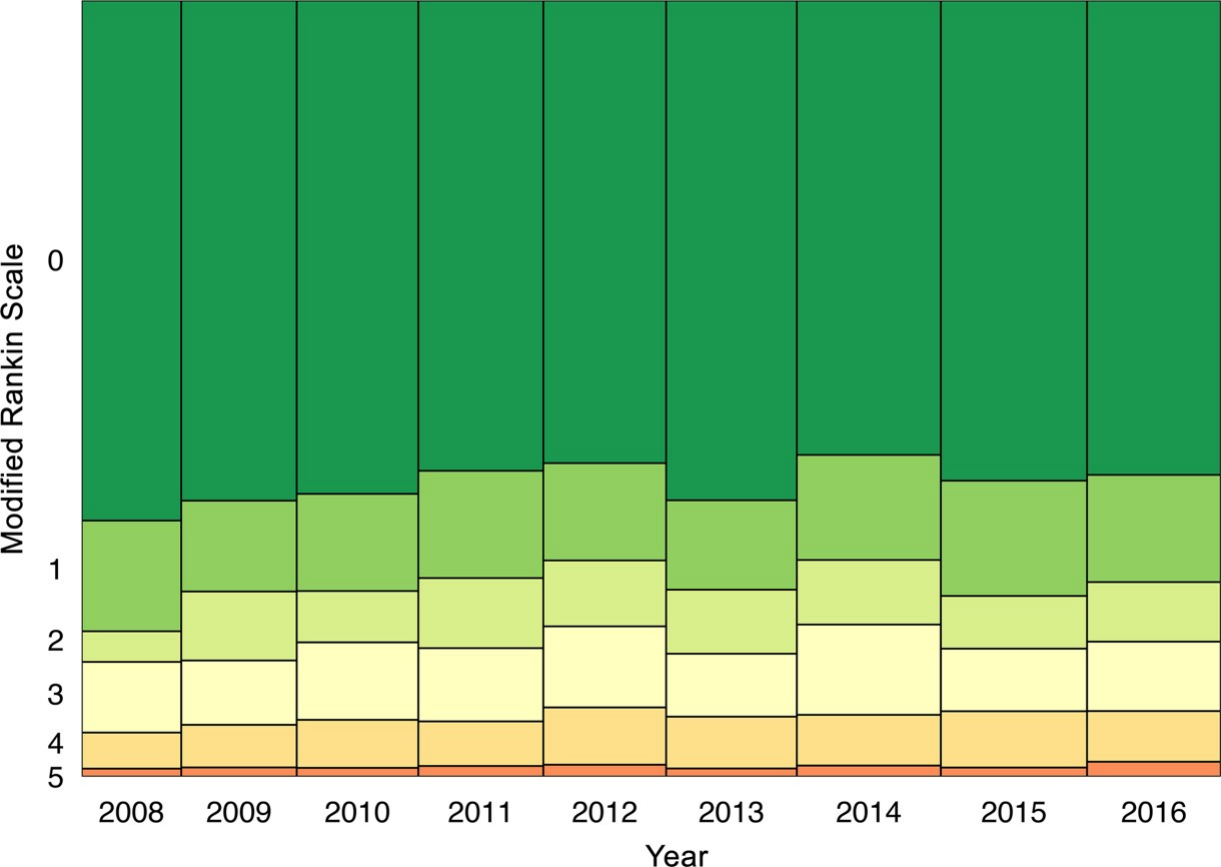
Pre-stroke Modified Rankin Scale. Pre-stroke Modified Rankin Scale (MRS) in patients with intracerebral hemorrhage registered in the Austrian Stroke Unit Registry between 2008 and 2016. The box width in the Mosaic plot indicates the number of available records in each year.

The median admission NIHSS score was 8, IQR 13. The percentage of high admission NIHSS scores (21–42) decreased over time while the rate of low admission NIHSS scores (0–5) increased (Fig 3). Moderate admission NIHSS scores (6–10, 11–20) showed no significant variation at the 5% significance level (Fig 3). The median pre-morbid mRS score was 0, IQR 0–1. No significant time trends were found in pre-morbid mRS scores (p = 0.921, Fig 4).

### Risk factors, etiologies and localizations of ICH

The prevalence of risk factors–in particular: hypertension (84.5%), diabetes mellitus (18.3%), previous stroke (18.3%), myocardial infarction (6.1%), hypercholesterolemia (33.8%), atrial fibrillation (22.2%), other cardiac disease (15.8%), peripheral arterial occlusive disease (4.6%), smoking (12.1%), regular alcohol consumption (9.5%), alcohol intoxication (0.8%), and etiologies including vascular malformations (5.0%), cerebral amyloid angiopathy (7.7%)–showed no significant variation at the 5% significance level in time trend analysis. Intracranial hemorrhage anatomical localization–deep (48.1%), lobar (41.2%), cerebellar (6.0%), brain stem (3.8%), ventricular (0.8%)–remained stable over the years (p = 1.0).

### Therapies

Recorded therapies included low dose heparin treatment, insulin therapy, antihypertensive agents, continuous intravenous therapy, assisted ventilation, tracheal intubation, nasogastric intubation, percutaneous gastrostomy, urinary catheterization, and surgical therapy. The evaluation of therapy measures revealed a significant decline of some interventions. The administration of low dose heparin decreased in favor of withholding heparin treatment (from 0.74 to 0.57, OR, 0.92; 95% CI, 0.89–0.95; p<0.001). The use of continuous intravenous therapy (from 0.31 to 0.24, OR, 0.96; 95% CI, 0.93–0.99; p = 0.022), nasogastric intubation (from 0.26 to 0.14, OR, 0.93; 95% CI, 0.90–0.96; p<0.001) and urinary catheterization (from 0.67 to 0.48, OR, 0.93; 95% CI, 0.91–0.96; p<0.001;) declined through the years. In contrast, no significant changes were observed in the frequency of insulin therapy (4.8%, p = 0.523), the use of antihypertensive agents (77.3%, p = 1.0), assisted ventilation (3.4%, p = 0.246), tracheal intubation (3.5%, p = 0.206), percutaneous gastrostomy (1.4%, p = 1.0), and surgical therapy (1.2%, p = 1.0). Considering low-dose heparinization, continuous intravenous therapy, and nasogastric intubation, we found a decreasing administration of treatment to patients suffering from a severe stroke (NIHSS 21–42) or patients of old age suffering from a severe stroke (age >80 to 120 years, NIHSS 21–42). Urinary catheterization declined most notably in elderly patients (>70 to 80 years and >80 years) with moderately severe stroke (NIHSS 11–20). While the administration of assisted ventilation did not significantly decline in general, a decrease was observed in patients suffering from a severe and moderately severe stroke (NIHSS 11–20 and 21–42).

### Complications

Brain edema (8.6%), epileptic seizures (2.8%), hydrocephalus (1.1%%), cardiac arrhythmia (1.6%), cardiovascular decompensation (2.7%), pulmonary embolism (0.3%), sepsis (0.8%), urinary tract infection (6.1%), pneumonia (11.3%), extracerebral hemorrhage (0.1%), and deep vein thrombosis (0.2%) remained unchanged at the 5% significance level in time trend analysis.

### Comparison of subjects with and without follow-up data

Follow-up data (mRS and mortality at 3 months) were available from 3582 subjects (53.4%, 51% male; median age 76.5 years, IQR 16.8 years, range 19–102 years). Patients with completed follow-up had more severe strokes (Table 1), higher pre-morbid MRS and admission NIHSS scores (Table 1), a greater prevalence of risk factors (Table 1), and higher complication rates (Table 2). Anatomical ICH localization did not significantly vary between patients with follow-up data and those without (p = 0.064). Time trends in the demographic and clinical parameters of patients with follow-up data were consistent with those described previously for the whole ICH study population: the proportion of ICH patients declined significantly over time (10.6% in 2008 vs 8.8% in 2012 vs 8.2% in 2016), with stable sex distribution and a shift towards older patients. The trend towards lower admission NIHSS scores was slightly less pronounced with a transition from high (21–42) to moderately high (11–20) scores and unchanged ratios of low and moderately low scores (0–5, 6–10). No significant time trends were found in pre-morbid mRS scores (p = 1.0) and ICH localization (p = 1.0). The prevalence of risk factors and complication rates showed no significant changes over time at the 5% significance level, while the same therapy measures that decreased in the general ICH study population–namely low dose heparin versus withholding heparin treatment (from 0.70 to 0.53, OR, 0.93; 95% CI, 0.89–0.97; p<0.001), continuous i.v. therapy (from 0.34 to 0.29, OR, 0.96; 95% CI, 0.93–0.99; p = 0.044), nasogastric intubation (from 0.32 to 0.22, OR, 0.93; 95% CI, 0.89–0.96; p<0.001), and urinary catheterization (from 0.74 to 0.60, OR, 0.93; 95% CI, 0.91–0.96; p<0.001)–also declined in the subgroup of patients with follow-up records.

**Table 1:** Base line characteristics of ICH patients with and without follow-up.

	follow-up	no follow-up	*p*-value
N	3582	3125	-
Age, median (Q_25_, Q_75_]^1^	76.5 (66.7, 83.5)	72.5 (61.7, 80.7)	< 0.001
Sex male (%)^2^	1823 (50.9)	1737 (55.6)	0.025
Pre-morbid MRS 4–5 (%)^2^	368 (10.3)	142 (4.5)	< 0.001
Admission NIHSS, median (Q_25_, Q_75_]^1^	13 (6, 20)	5 (2, 9)	< 0.001
Hypertension (%)^2^	3039 (85.3)	2586 (83.6)	< 0.001
Diabetes mellitus (%)^2^	655 (18.4)	564 (18.2)	< 0.001
Previous stroke (%)^2^	707 (19.8)	514 (16.6)	< 0.001
Myocardial infarction (%)^2^	238 (6.7)	168 (5.4)	< 0.001
Hypercholesterolemia (%)^2^	1201 (33.7)	1049 (33.9)	< 0.001
Atrial fibrillation (%)^2^	866 (24.3)	610 (19.7)	< 0.001
Other cardiac disease (%)^2^	617 (17.3)	436 (14.1)	< 0.001
Peripheral arterial occlusive disease (%)^2^	170 (4.8)	134 (4.3)	< 0.001
Smoking (%)^2^	377 (10.6)	427 (13.8)	< 0.001
Regular alcohol consumption (%)^2^	302 (8.5)	333 (10.8)	< 0.001
Vascular malformations (%)^2^	152 (4.2)	186 (6)	0.284
Cerebral amyloid angiopathy (%)^2^	285 (8)	234 (7.5)	1.0

^1^ Kruskal-Wallis test

^2^ Chi-Square test of independence.

ICH, intracranial hemorrhage; Q_25_, 25% quartile; Q_75_, 75% quartile; MRS, Modified Rankin Scale; NIHSS, National Institutes of Health Stroke Scale. The percentages given are based on the available records in a group.

**Table 2:** Complication rates in ICH patients with and without follow-up data.

	follow-up	no follow-up	*p*-value
N	3582	3125	-
Brain edema (%)^1^	539 (15.1)	38 (1.2)	< 0.001
Epileptic seizures (%)^1^	100 (2.8)	88 (2.8)	1.0
Hydrocephalus (%)^1^	64 (1.8)	10 (0.3)	< 0.001
Cardiac arrhythmia (%)^1^	84 (2.3)	22 (0.7)	< 0.001
Cardiovascular decompensation (%)^1^	155 (4.3)	23 (0.7)	< 0.001
Pulmonary embolism (%)^1^	10 (0.3)	9 (0.3)	1.0
Sepsis (%)^1^	44 (1.2)	12 (0.4)	0.031
Urinary tract infection (%)^1^	244 (6.8)	164 (5.3)	1.0
Pneumonia (%)^1^	553 (15.5)	203 (6.5)	< 0.001
Extracerebral hemorrhage (%)^1^	6 (0.2)	4 (0.1)	1.0
Deep vein thrombosis (%)^1^	7 (0.2)	5 (0.2)	1.0

^1^ Chi-Square test of independence.

ICH, intracranial hemorrhage. The percentages given are based on the available records in a group.

### Functional outcome and mortality at 3 months

**Fig 5:**
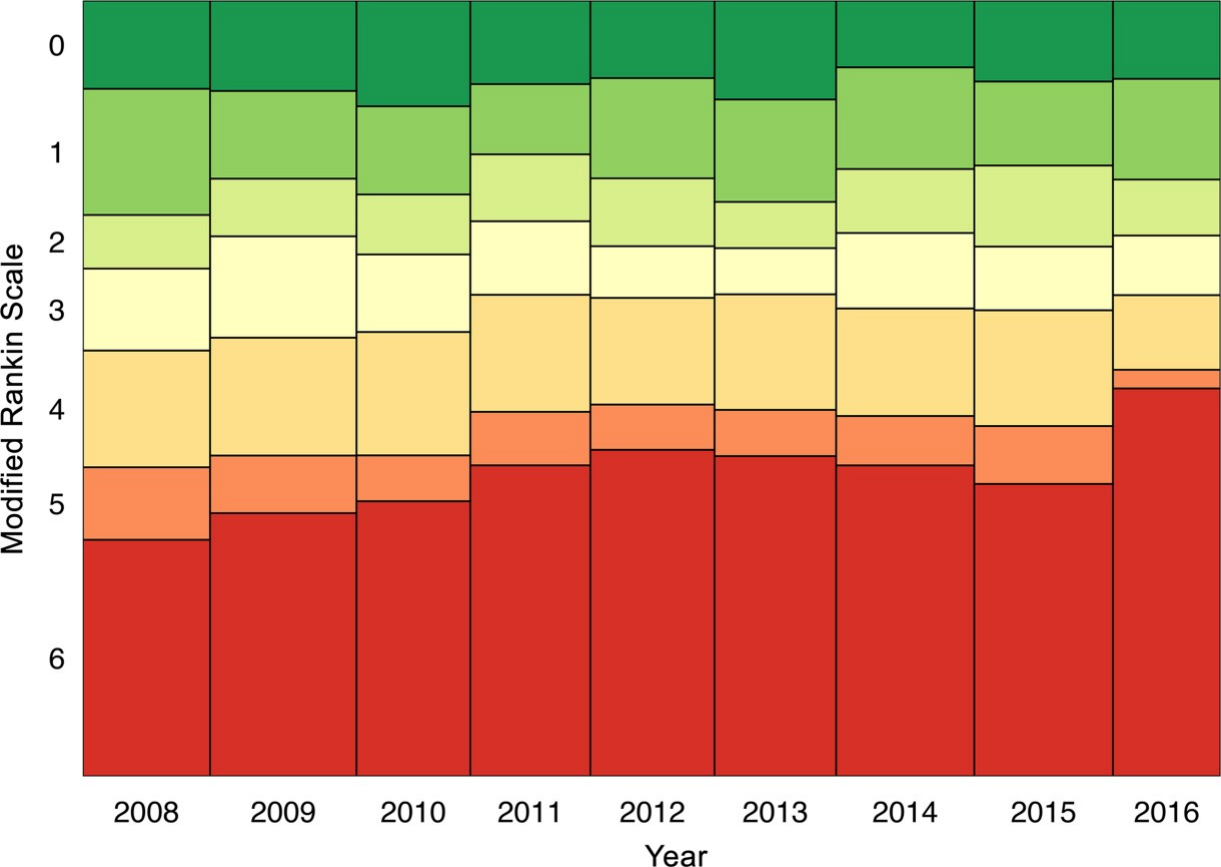
Follow-Up Modified Rankin Scale at three months. Follow-Up Modified Rankin Scale (MRS) at three months in patients with intracerebral hemorrhage and information on follow-up outcome registered in the Austrian Stroke Unit Registry between 2008 and 2016. MRS 6 indicates death at 90 days follow-up. The box width in the Mosaic plot indicates the number of available records in each year.

Follow-up data were available for 3582 subjects. At 3-months-follow-up, the percentages of mRS scores 0–3 versus 4–6 showed no significant changes over the whole study period between 2008 and 2016 (p = 1.0). However, there was a significant increase of patients with mRS score 6 (representing death/mortality) in comparison to mRS scores 0–5 (OR, 1.06; 95% CI, 1.02–1.11; p = 0.003) over time. A steady increase of follow-up mortality between 2008 and 2012 was followed by a slight decline between 2012 and 2015, and a sharp rise in 2016 (Fig 5). In multivariate analyses, age >80 years was associated with unfavorable outcome and increased mortality (adjusted OR, 5.17; 95% CI, 2.23–11.97; p<0.001; and adjusted OR, 3.17; 95% CI, 1.4–7.18; p = 0.008; Table 3) without time interaction. Likewise, moderately high and high admission NIHSS scores (11–20, 21–42) were associated with unfavorable outcome (adjusted OR, 7.64; 95% CI, 2.49–23.46; p<0.001; and adjusted OR, 68.23; 95% CI, 2.45–1897.12; p = 0.042) and increased mortality (adjusted OR, 9.30; 95% CI,3.54–24.39; p<0.001; and adjusted OR, 39.08; 95% CI,8.39–182; p<0.001; Table 3). Moreover, groups of moderate admission NIHSS scores (6–10 and 11–20) were associated with increased mortality also when entered as interaction with time, independently of all other variables (adjusted OR, 1.20 and 1.22; 95% CI, 1.07–1.35 and 1.08–1.38; p<0.001). No interaction with time was seen in those with low (0–5) and high (21–42) admission NIHSS scores (p = 1.0).

**Table 3:** Multivariate regression model to predict follow-up mortality at 3 months.

	Odds ratio	95% Confidence Interval	*p*-value
Age (50,70]	1.05	0.47 to 2.32	1.0
Age (70,80]	1.71	0.78 to 3.80	1.0
Age (80,120]	3.17	1.40 to 7.18	0.008
Admission NIHSS 0–5^1^	-	-	-
Admission NIHSS 6–10	1.82	0.69 to 4.78	1.0
Admission NIHSS 11–20	9.30	3.54 to 24.39	< 0.001
Admission NIHSS 21–42	39.08	8.39 to 182.0	< 0.001
Admission NIHSS 0–5 * Year	1.03	0.91 to 1.17	1.0
Admission NIHSS 6–10 * Year	1.20	1.07 to 1.35	< 0.001
Admission NIHSS 11–20 * Year	1.22	1.08 to 1.38	< 0.001
Admission NIHSS 21–42 * Year	1.16	0.88 to 1.52	1.0
Heparin treatment^1^	-	-	-
Continuous i.v. therapy^1^	-	-	-
Nasogastric intubation	1.73	1.07 to 2.79	0.176
Urinary catheterization	2.28	1.57 to 3.31	< 0.001

^1^ Eliminated by the regression model and not associated with follow-up mortality.

Patients with moderate admission NIHSS scores of 6–10 and 11–20 showed an increasing mortality risk over the years independent of any other variables

* for interaction between variables

### Subjects with pre-stroke mRS ≤ 3

After excluding severely premorbid subjects with pre-stroke mRS scores of 4 and 5, an additional assessment of patients with pre-stroke mRS scores ≤ 3 showed slightly lower median admissions NIHSS scores than in the general study population (median 7, IQR 12 versus median 8, IQR 13) and a lower percentage of patients with previous stroke (16.2% versus 18.3%). The baseline characteristics remained otherwise unchanged. With regard to risk factors, etiologies, localizations of ICH, and complications, no significant variations were found. Functional outcome and mortality at 3 months revealed the same significant trends as in the general study population–most notably and independently of all other variables, an increased mortality in patients with moderate admission NIHSS scores (6–10 and 11–20) when entered as interaction with time (adjusted OR; 1.21; 95% CI 1.1–1.41; p<0.001; and adjusted OR 1.24, 95% CI 1.1–1.41; p<0.001). Likewise, the observed trends in therapies were confirmed in this subset of patients.

## Discussion

Data from the Austrian Stroke Unit Registry suggest that mortality in acute ICH patients treated at stroke units increased significantly between 2008 and 2016, despite largely unchanged risk factors, declining admission NIHSS scores, and stable complication rates. Over the 9 years of the study, patients with acute ICH became older and had milder strokes. The ratio of ICH among all acute stroke admissions to Austrian stroke units declined significantly from 6.3% in 2008 to 4.7% in 2016, without changes in the nationwide patient admission and distribution system. This is in line with earlier observations from the Austrian Stroke Unit Registry [19] and other populations [1,6,9,13]. At 5.6% mean, the overall proportion of ICH patients was slightly lower in our acute stroke study population than in previous reports [1,2,5]. This may be due to the fact that the ASUR has collected data exclusively from patients admitted to stroke units, thus omitting patients admitted primarily to neurosurgery departments or general intensive care units. The functional outcome showed no significant time trends except for mortality: in 2016, one in two patients (50.0%) treated for acute ICH died within 90 days from admission, compared to one in three patients (30.5%) in 2008. This trend may be mostly explained by an increase in patients aged ≥ 70 years. We found that age ≥ 80 years was associated with increased follow-up mortality, and it has previously been reported that patients ≥ 75 years of age experienced no improvement [12] or even deterioration [20] in ICH. However, the multivariable analyses showed that patients with moderate admission NIHSS scores (6–10 and 11–20) are over time at greater likelihood of dying, independent of age and all other assessed factors known to contribute to mortality. The reasons for this increase in the mortality risk are unclear. Interestingly, we observed changes in some therapies of acute ICH patients in parallel over the same time. It has previously been shown that early care limitations including do not resuscitate (DNR) orders are associated with short- and long-term mortality after ICH, and the reluctance of clinicians to offer aggressive care in the absence of evidence-based curative therapy for severe ICH could be reaffirmed in the sense of a self-fulfilling prophecy [2,21–23]. While data on DNR orders were not available in our study, the observed decline of certain interventions may hypothetically point towards a growing willingness to withdraw from care. When considering therapies such as continuous intravenous therapy, nasogastric intubation, urinary catheterization, and assisted ventilation, we found a decreasing use of these treatments in patients with severe strokes or patients of old age with severe strokes. These trends were confirmed to be significant by detailed exploration of the data, however, these four-way interactions could not be included into the logistic regression model for mortality due to masking effects. Interpretations linking declining trends in therapies to the increased mortality may therefore only be postulated with caution as a hypothesis. Studies on care limitations in patients with ICH have shown that practitioners tend to be overly pessimistic in predicting outcome based upon data available at the time of presentation [22], that aggressive care after ICH can favorably impact functional outcome and survival [21,24], and that individuals in the worst initial prognostic category can have meaningful recovery when offered full aggressive care [21]. Older age, which is also associated with worse prognosis in ICH, has been shown to increase the likelihood of a DNR decision [25], a particularly noteworthy finding in view of the increasing age of patients. Death may not always be an undesired outcome in many dire circumstances, particularly in patients of old age with severe strokes. An increasing mortality rate in patients with moderate admission NIHSS scores over time, however, gives cause for concerns.

Several limitations must be named for interpretation of the results. First, in Austria, two thirds of all strokes admitted to hospitals are treated at stroke-units [15,19]. Data of the ASUR can thus be seen as representative for acute stroke patients in Austria [19]. However, the ASUR does not include ICH patients admitted primarily to neurosurgery departments or general intensive care units. Our results are therefore limited to a specific population of ICH patients treated at stroke units, omitting those admitted directly or transferred to neurosurgical or intensive care departments. This may introduce significant bias, as ICH patients with better prognosis are more likely to be treated surgically or in intensive care. Nonetheless, against the background of declining admission NIHSS scores in ICH stroke unit patients, it is surprising that the mortality rate in conservative stroke treatments seems to increase over the years. This contradicts previous studies on the clearly positive effects of a dedicated stroke unit care on ICH outcome [26]. Second, the analyses are based on specific hospital data and thus do not allow conclusions on incidence or risk factor changes in the general population or on their relative importance for stroke risk. The differences in patients with follow-up data as compared to those without follow-up may be partially explained by the fact that all acutely deceased patients automatically receive follow-up records. This may introduce bias with overrepresentation of more severe cases resulting in higher mortality. Nevertheless, *time trends* in mortality should not be affected by this bias as the system of follow-up recording did not change over time. Additionally, this presumption is underlined by the fact that time trends for other variables did not vary significantly between patients with follow-up data and the general ICH study population. Despite the above-mentioned limitations, the strength of this prospective register is the uniform collection of nationwide stroke unit data over a 9 years period, which allows the assessment of major temporal trends in stroke patients' characteristics and inferences for the future management of acute stroke patients.

## Conclusion

In line with findings from earlier years [19], our data further support the assumption that acute stroke management will have to focus increasingly on patients with ICH. The observed rise in mortality of ICH patients treated on stroke units is a novel finding and may be reason for concern. It is in conflict with previous reports of stable mortality rates over the years in ICH and decreasing mortality rates in ischemic stroke. Nevertheless, our observation should clearly be seen as a call for action in stroke unit management for this devastating condition.

## Supporting information

S1 STROBE checklist (DOC)
