# Supplementary material for: Temporal trends in intracerebral hemorrhage: Evidence from the Austrian Stroke Unit Registry
Source: PLoS One. 2019 Nov 20;14(11):e0225378. doi: 10.1371/journal.pone.0225378 (PMC6867701; doi:10.1371/journal.pone.0225378)
Supplement: S1 STROBE checklist — (DOC) [file pone.0225378.s001.doc]

STROBE Statement—Checklist of items that should be included in reports of ***cohort studies***

|  | Item No | Recommendation |
| --- | --- | --- |
| **Title and abstract** | 1 | (*a*) Indicate the study’s design with a commonly used term in the title or the abstract  page 5 "The observations from this large retrospective database cohort study (...)." |
|  |  | (*b*) Provide in the abstract an informative and balanced summary of what was done and what was found  page 4 |
| Introduction | | |
| Background/rationale | 2 | Explain the scientific background and rationale for the investigation being reported  page 6 "Intracerebral hemorrhages (ICH) make up approximately 10-20% of all strokes in western high-income countries, however, the burden of stroke mortality and morbidity associated with ICH is disproportionately high. While the incidence of ischemic stroke decreased in recent decades, limited data exists on the incidence of ICH and available reports came to conflicting results." |
| Objectives | 3 | State specific objectives, including any prespecified hypotheses  page 6 "The aim of this analysis was to examine trends in ICH incidence, severity, complications, therapy and outcome of ICH from 2008 to 2016 in subjects registered in the Austrian Stroke Unit Registry (ASUR)." |
| Methods | | |
| Study design | 4 | Present key elements of study design early in the paper  pages 6-8 |
| Setting | 5 | Describe the setting, locations, and relevant dates, including periods of recruitment, exposure, follow-up, and data collection  pages 6-8 |
| Participants | 6 | (*a*) Give the eligibility criteria, and the sources and methods of selection of participants. Describe methods of follow-up  pages 6-8 |
|  |  | (*b*) For matched studies, give matching criteria and number of exposed and unexposed  [n/a] |
| Variables | 7 | Clearly define all outcomes, exposures, predictors, potential confounders, and effect modifiers. Give diagnostic criteria, if applicable  pages 6–8 |
| Data sources/ measurement | 8* | For each variable of interest, give sources of data and details of methods of assessment (measurement). Describe comparability of assessment methods if there is more than one group  pages 6–8 |
| Bias | 9 | Describe any efforts to address potential sources of bias  [n/a] |
| Study size | 10 | Explain how the study size was arrived at  pages 6–8 |
| Quantitative variables | 11 | Explain how quantitative variables were handled in the analyses. If applicable, describe which groupings were chosen and why  pages 6–8 |
| Statistical methods | 12 | (*a*) Describe all statistical methods, including those used to control for confounding  page 7 |
|  |  | (*b*) Describe any methods used to examine subgroups and interactions  page 7 |
|  |  | (*c*) Explain how missing data were addressed  pages 14ff "Comparison of subjects with and without follow-up data" |
|  |  | (*d*) If applicable, explain how loss to follow-up was addressed  pages 14ff "Comparison of subjects with and without follow-up data" |
|  |  | (*e*) Describe any sensitivity analyses  [n/a] |
| Results | | |
| Participants | 13* | (a) Report numbers of individuals at each stage of study—eg numbers potentially eligible, examined for eligibility, confirmed eligible, included in the study, completing follow-up, and analysed  pages 8, 14ff |
|  |  | (b) Give reasons for non-participation at each stage  [n/a] |
|  |  | (c) Consider use of a flow diagram  [n/a] |
| Descriptive data | 14* | (a) Give characteristics of study participants (eg demographic, clinical, social) and information on exposures and potential confounders  pages 8ff, 16 |
|  |  | (b) Indicate number of participants with missing data for each variable of interest  pages 16, 17 |
|  |  | (c) Summarise follow-up time (eg, average and total amount)  [n/a] |
| Outcome data | 15* | Report numbers of outcome events or summary measures over time  pages 8ff |
| Main results | 16 | (*a*) Give unadjusted estimates and, if applicable, confounder-adjusted estimates and their precision (eg, 95% confidence interval). Make clear which confounders were adjusted for and why they were included  page 19 |
|  |  | (*b*) Report category boundaries when continuous variables were categorized  page 7 "The subjects were divided into 4 age groups (...) and admission NIHSS scores were split into 4 categories (...). The functional outcome at 3 months was dichotomized into favorable outcome (mRS 0-3) (...) versus unfavorable outcome (mRS 4-6) (...)." |
|  |  | (*c*) If relevant, consider translating estimates of relative risk into absolute risk for a meaningful time period  [n/a] |
| Other analyses | 17 | Report other analyses done—eg analyses of subgroups and interactions, and sensitivity analyses  page 14, "Comparison of subjects with and without follow-up data" |
| Discussion | | |
| Key results | 18 | Summarise key results with reference to study objectives  pages 20ff, 24 |
| Limitations | 19 | Discuss limitations of the study, taking into account sources of potential bias or imprecision. Discuss both direction and magnitude of any potential bias  pages 22–23 |
| Interpretation | 20 | Give a cautious overall interpretation of results considering objectives, limitations, multiplicity of analyses, results from similar studies, and other relevant evidence  pages 20ff |
| Generalisability | 21 | Discuss the generalisability (external validity) of the study results  pages 20ff |
| Other information | | |
| Funding | 22 | Give the source of funding and the role of the funders for the present study and, if applicable, for the original study on which the present article is based  page 3 |

*Give information separately for exposed and unexposed groups.

**Note:** An Explanation and Elaboration article discusses each checklist item and gives methodological background and published examples of transparent reporting. The STROBE checklist is best used in conjunction with this article (freely available on the Web sites of PLoS Medicine at http://www.plosmedicine.org/, Annals of Internal Medicine at http://www.annals.org/, and Epidemiology at http://www.epidem.com/). Information on the STROBE Initiative is available at http://www.strobe-statement.org.
